# Perrault Syndrome Diagnosis in a Patient Presenting to Her Primary Care Provider with Secondary Amenorrhea

**DOI:** 10.1155/2019/9865281

**Published:** 2019-06-02

**Authors:** Leah May Roberts, Bruce Carnivale

**Affiliations:** Department of Obstetrics, Gynecology and Reproductive Sciences, Temple University Hospital, 3401 N Broad Street, Philadelphia, PA 19140, USA

## Abstract

Perrault syndrome is a rare autosomal recessive genetic disorder characterized by sensorineural hearing loss and female gonadic eukaryotic dysgenesis. Here, we report the case of a 20-year-old female that presented to her internal medicine physician after suffering from secondary amenorrhea. After multiple negative pregnancy tests done with primary care physicians, a further evaluation by an internal medicine specialist showed an elevated FSH and LH as well as a small uterus and streak ovaries on transabdominal and transvaginal ultrasound. She was referred to the OB-GYN service, gonadal dysgenesis was diagnosed, and proper treatment was initiated. We intend to highlight this presentation of ovarian dysfunction and provide guidance for the proper diagnosis and management of the said disorder.

## 1. Introduction

Perrault syndrome, first described by Perrault in 1951, is an inherited autosomal recessive congenital disorder that is characterized by sensorineural hearing loss in both genders and female gonadal dysgenesis [1]. Perrault initially described these patients as variants of Turner syndrome; however, further evaluation of these patients as adults by Josso showed they had a normal 46XX karyotype [2, 3]. There are two types: type I, which is static without neurologic disease, and type 2, which includes progressive neurologic disease [1]. The symptoms of type two include absent tendon reflexes, nystagmus dysarthria, cognitive impairment, scoliosis, and cerebellar atrophy [4, 5]. It is difficult to assess total number of cases, as most men with unaffected sisters are less likely to be appropriately diagnosed, as there are over 400 syndromic forms of deafness. Currently, the total number of diagnosed cases is approximately one hundred [6, 7].

## 2. Case Presentation

20yo G0P0 female presented with what was thought to be secondary amenorrhea to her internal medicine physician after multiple negative pregnancy tests. She stated she had one year of normal monthly menses at 12-13, but had no periods since then. She had mittelschmerz-like cramping pelvic pain monthly without menses. Past medical history was notable for congenital sensorineural deafness. No other members of her family were noted to be deaf. All visits were completed with an American Sign Language interpreter. She had a negative surgical history. Her social history was significant for alcohol and marijuana usage. Review of system was positive for fatigue and pelvic pain only. She was noted to be 1.676m and weighed 62.6kg (BMI 22.3 kg/m^2^). Her blood pressure was 93/64 mmHg and her pulse was 75 beats per minute. No abnormalities were noted on physical exam. Specifically no webbing was noted in her neck, and no hyperpigmentation of the skin was noted. A screening breast exam noted some development consistent with her ectomorphic body type.

**Figure 1:**
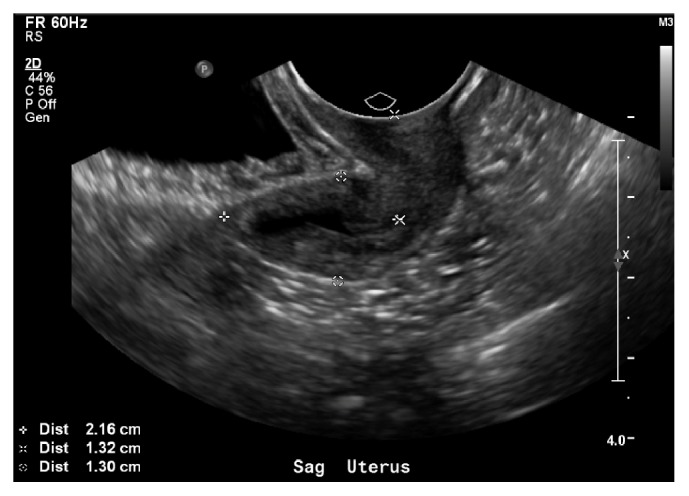
Transvaginal ultrasound of uterus.

**Figure 2:**
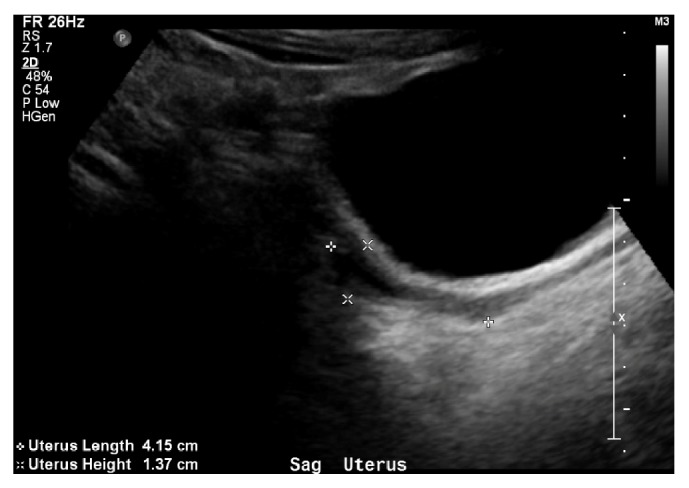
Transabdominal ultrasound of uterus.

A full set of labs were sent by her internal medicine attending physician to help diagnose her secondary amenorrhea including CBC, CMP, TSH, FSH, LH, DHEA, testosterone, prolactin, and hCG, as well as complete pelvic ultrasound. She was noted to have normal testosterone, DHEA, prolactin, TSH, and a negative b-HCG; however, she had elevated FSH (145.4) and LH (52.1). Pelvic exam showed a small uterus, measuring 4.1x1.4x2.2cm (see Figures 1 and 2), and streak ovaries, with a small amount of fluid in the endometrial canal and a stripe of 2mm. Right ovary was 0.7x1.1x1cm and left ovary was 0.6x1x0.6cm. At this time she was referred to a gynecologist for care.

At the time of initial gynecology consultation, she stated she had one single episode of vaginal bleeding at age 12 and did not have any further menses. This was in stark contrast to what had been previously reported and was elicited by an in depth review of systems. This is noted to be more consistent with her laboratory and ultrasound findings, and thereby she was diagnosed with primary amenorrhea. She was noted to have normal olfactory testing. On exam by OB-GYN she was actually noted to have Tanner III breast and pubic hair development. She did complain of vaginal dryness, especially with intercourse.

She underwent karyotype, which was 46XX, and was diagnosed with hypergonadotropic hypogonadism with hearing loss, which is consistent with Perrault syndrome, gonadal dysgenesis XX type. She was started on OCPs at this time for protection for osteoporosis and heart disease.

Due to the cost of testing and her insurance coverage, she has not yet had her specific molecular diagnosis made. At this time, as it will not change her management, she does not desire to have genetic testing performed.

## 3. Discussion

This case was particularly interesting as the patient initially presented to primary care providers who initially sent multiple pregnancy tests and attempted to reassure the patient. However, when she went to an internal medicine provider, a thorough work-up for amenorrhea was performed and gonadal dysgenesis was diagnosed. Although she in fact had primary amenorrhea, she initially stated that she previously had a regular period for a year. A thorough physical examination, laboratory evaluation, and ultrasound performed by both her internal medicine and OB-GYN physicians led to the proper diagnosis of Perrault syndrome. The early development of certain sexual characteristics can be misleading.

Age of onset of both hearing loss and ovarian dysfunction can be variable. Hearing loss ranges from being congenital in onset to beginning in early childhood. Ovarian dysfunction ranges from presentation with primary amenorrhea to primary ovarian insufficiency. Median age of diagnosis in the literature is 22 [4].

Genetic diagnosis cannot always be established; however, biallelic pathogenic variants in six known genes provide definitive diagnosis. Approximately 60% of individuals who have undergone genetic testing have not received a molecular diagnosis. Genes identified include CLPP, ERAL1, HARS2, HSD17B4, LARS2, and TWNK [7, 8]. All of these known variations are associated with dysfunction of the mitochondria [9].

Treatment of hearing loss depends on level of loss and age at time of loss and ranges from additional educational resources to cochlear implantation. This is generally accomplished in close collaboration with an ENT and an audiologist as well as the child's school system. Routine surveillance for progression in hearing loss is important [7].

Treatment of ovarian dysfunction also depends on age of diagnosis and level of loss of function. If the patient has not undergone puberty, estrogen replacement can be given to facilitate this process. If the patient has undergone puberty, the maintenance of bone health with oral contraceptive pills until approximately age fifty is appropriate. This also reduces the risks of cardiovascular disease [7]. Infertility treatments generally include egg donor and gestational surrogate, as not only is there premature ovarian failure, but the uterus is often rudimentary and prepubertal [7]. One case in the literature describes a woman who had delivered two sons before her premature ovarian failure was diagnosed on presentation for secondary amenorrhea at twenty-two [8].

Differential diagnosis includes Turner syndrome, as approximately 1/2 of Turner patients have some degree of hearing loss [10]. Karyotype analysis excludes this diagnosis. Swyer Syndrome also needs to be considered in all cases of primary gonadal dysgenesis—given the high incidence of malignancy—but this again is ruled out by karyotype testing (46 XY). Several other molecular diagnoses can be distinguished from Perrault syndrome by analysis of these known genes including FMR1 expansions, BPES, and polyglandular autoimmune syndromes types 1 and 2 [7, 11].

In conclusion, physicians should be aware that young female patients may come with little understanding of their sexual development and cycle, thereby providing misleading information. Our patient truly believed she did “have a cycle” that just stopped. As such, the importance of a thorough history, complete detailed exam, and patient education about female growth and sexual health cannot be overstated. Also, both primary care physicians and gynecologists should be familiar with and be able to provide resources for rare genetic diagnosis.
